# Supplementary material for: Characterization of Wingbeat Frequency of Different Taxa of Migratory Insects in Northeast Asia
Source: Insects. 2022 Jun 3;13(6):520. doi: 10.3390/insects13060520 (PMC9224674; doi:10.3390/insects13060520)
Supplement: Supplementary file 1 [file insects-13-00520-s001.zip › Supplementary materials/Supplemental Figures.pdf]

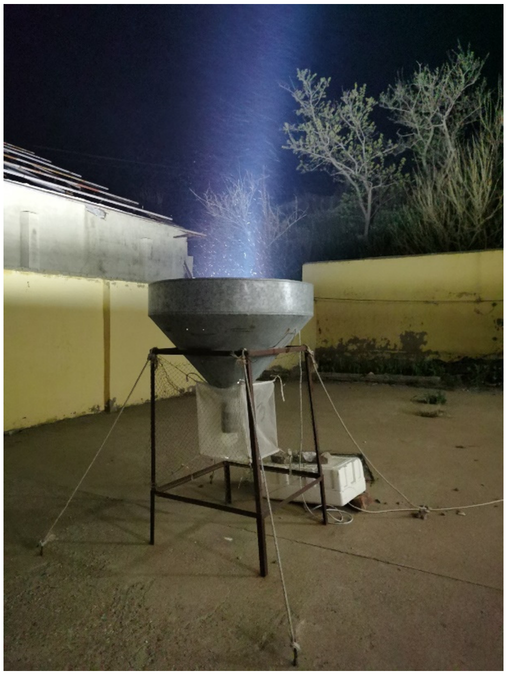

Figure S1. Picture of the searchlight trap.

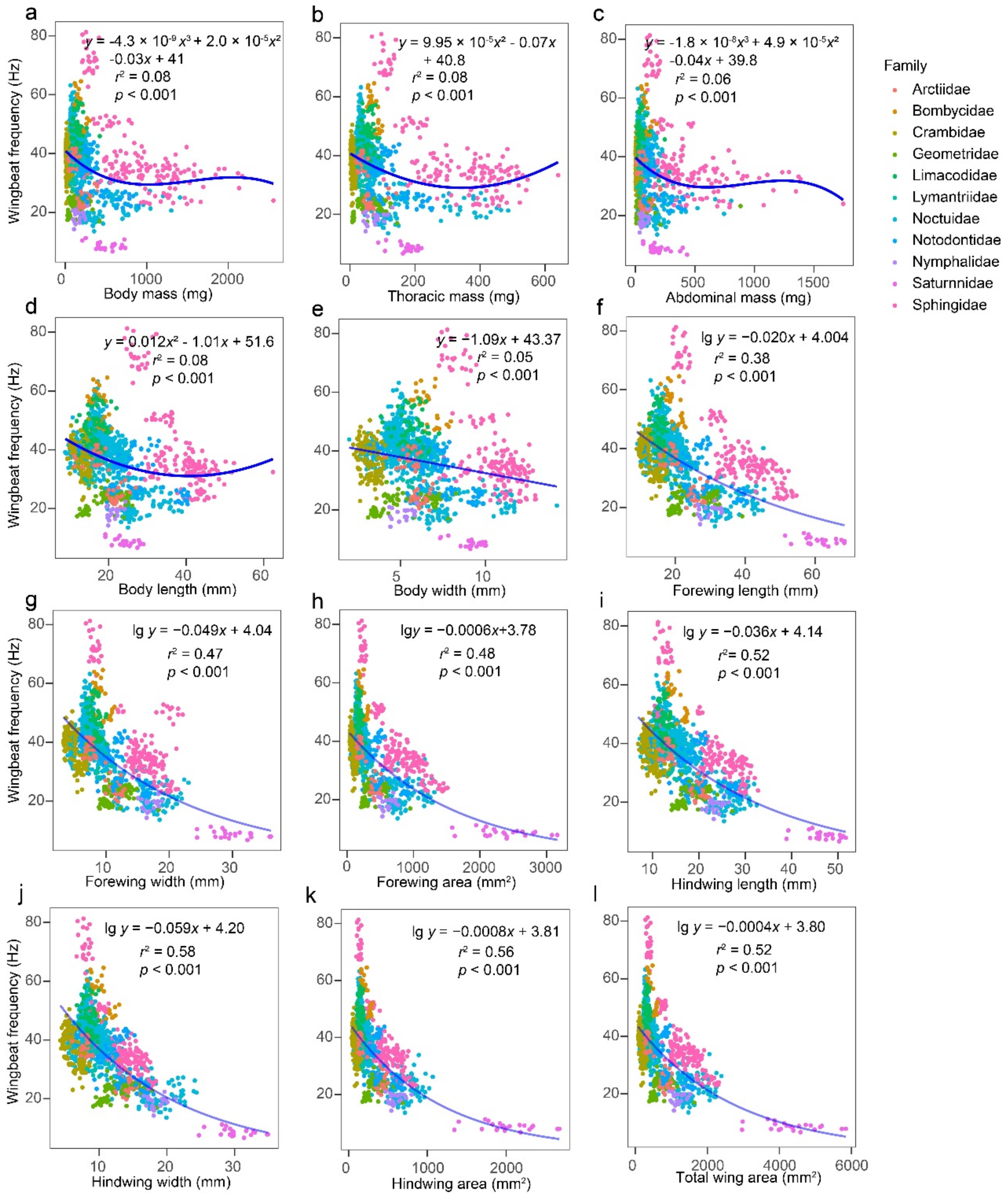

Figure S2. Relationship between wingbeat frequency and morphological variables (a) body mass, (b) thoracic mass, (c) abdominal mass, (d) body length, (e) body width, (f) fore-wing length, (g) forewing width, (h) forewing area, (i) hindwing length, (j) hindwing width, (k) hindwing area, and (l) total wing area, for all tested lepidopterans. $N = 1344$ individuals.

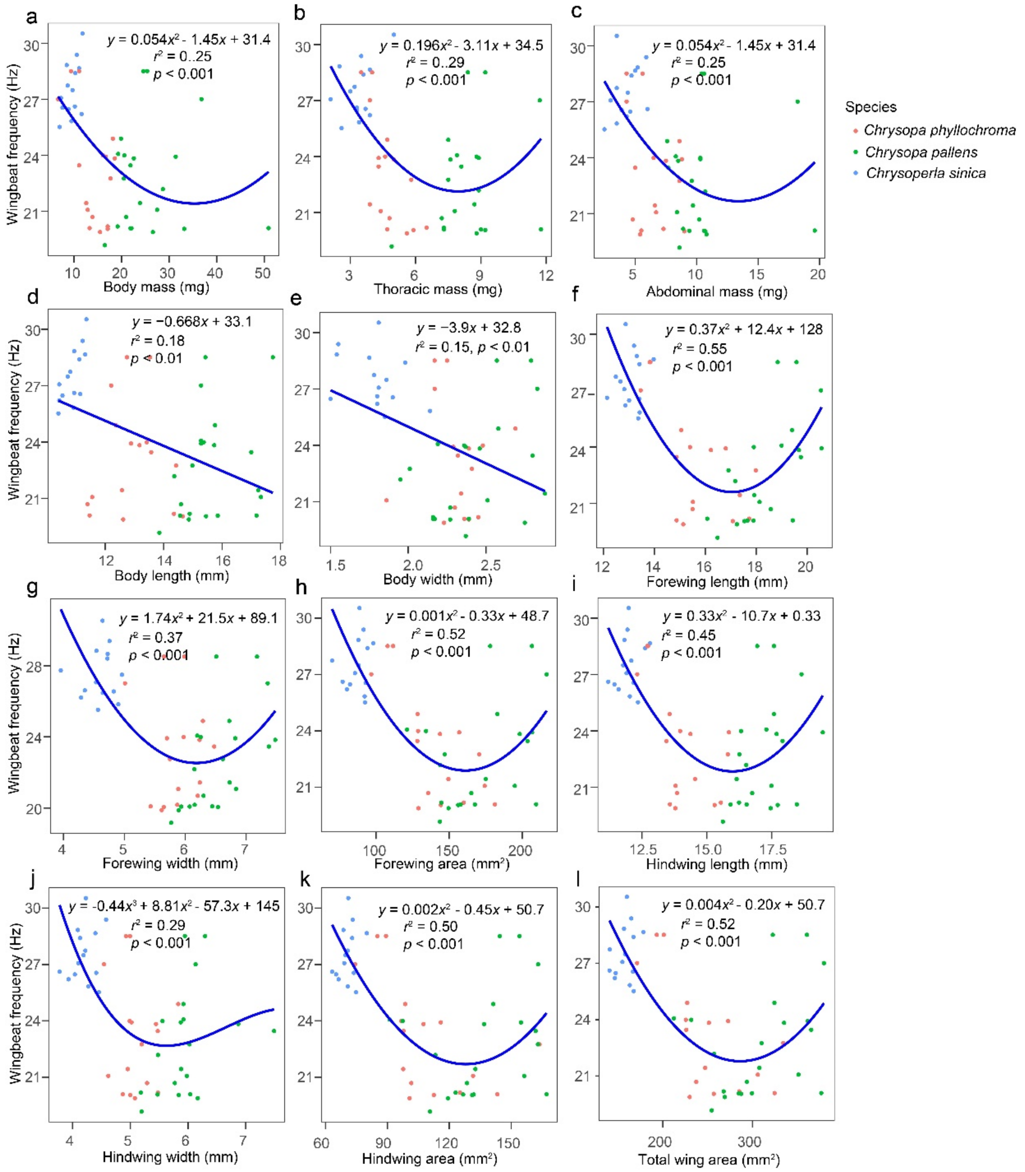

Figure S3. Relationship between wingbeat frequency and morphometrics (a) body mass, (b) thoracic mass, (c) abdominal mass, (d) body length, (e) body width, (f) fore-wing length, (g) forewing width, (h) forewing area, (i) hindwing length, (j) hindwing width, (k) hindwing area, and (l) total wing area, of all test neuropterans. $N = 51$ individuals.
